# Use of LAMP for Assessing *Botrytis cinerea* Colonization of Bunch Trash and Latent Infection of Berries in Grapevines

**DOI:** 10.3390/plants9111538

**Published:** 2020-11-11

**Authors:** Melissa Si Ammour, Eleonora Castaldo, Giorgia Fedele, Vittorio Rossi

**Affiliations:** 1 Department of Sustainable Crop Production (DI.PRO.VES.), Università Cattolica del Sacro Cuore, Via E. Parmense 84, 29122 Piacenza, Italy; melissa.siammour@unicatt.it (M.S.A.); giorgia.fedele@unicatt.it (G.F.); 2 Enbiotech s.r.l., Via Quarto dei Mille 6, 90129 Palermo, Italy; e.castaldo@enbiotech.eu

**Keywords:** loop-mediated isothermal amplification, real-time monitoring, on-site testing, crude extract, Botrytis bunch rot

## Abstract

A real-time loop-mediated isothermal amplification (LAMP) assay was evaluated for the detection of *Botrytis cinerea* in grapevine bunch trash, immature berries, and ripening berries. A simple method for the preparation of crude extracts of grape tissue was also developed for on-site LAMP analysis. When tested with 14 other fungal species frequently found in grapevines, the LAMP assay was specific and sensitive to a *B. cinerea* DNA quantity of 0.1 ng/µL. The sensitivity was further tested using bunch trash samples with *B. cinerea* colonization levels between 6 and 100% and with bulk-berry samples composed of 4 pathogen-free berries or 4 berries among which 25 to 100% had been inoculated with *B. cinerea*. The LAMP assay detected the lowest *B. cinerea* colonization level tested in bunch trash and in immature and mature berries in less than 20 min. In single-berry experiments, LAMP amplified *B. cinerea* DNA from all artificially inoculated individual immature and mature berries. No amplification occurred in *B. cinerea*-free material. The real-time LAMP assay has the potential to be used as a rapid on-site diagnostic tool for assessing *B. cinerea* colonization in bunch trash and *B. cinerea* latent infections in berries, which represent critical stages for decision-making about disease management.

## 1. Introduction

*Botrytis cinerea* Pers. Fr. (teleomorph *Botryotinia fuckeliana* (de Bary) Whetzel) is a ubiquitous fungus that grows both parasitically and saprophytically on more than 230 plant species [1]. On grapevines (*Vitis vinifera* L.), the fungus causes Botrytis bunch rot (BBR), a disease of major economic importance [2,3].

*Botrytis cinerea* infects grapevines via several pathways [4], with one infection window between flowering to young cluster (corresponding to growth stages 53 and 73 [5], respectively), and a second infection window between veraison to berry maturity (growth stages 79 and 89, respectively). In the early season, conidia infect flowers through the styles, ovules, stamens, or petals, and infect young berries via the pedicel [4]. These infections cause blossom blight and the saprophytic colonization of “bunch trash” consisting of calyptras, tendrils, dead stamens, aborted flowers and berries [4,6]. Under favorable conditions, the saprophytic mycelium produces abundant conidia on the colonized bunch trash, which is considered a major source of inoculum for late-season infections [7,8,9,10]. In young berries, the fungus develops latent infections, and the berries remain symptomless until the fungus reactivates after veraison and causes berry rot [11,12,13]. After veraison, new infections on berries can be caused either by airborne conidia or by contact with the aerial mycelium growing from adjacent rotted berries (berry-to-berry infections) [4,14].

Control of *B. cinerea* mainly relies on the application of plant protection products (mainly fungicides but also botanicals and biocontrol agents [15,16,17,18]) at four vine growth stages, which correspond to the periods of risk for the main infection pathways [19,20]. Plant protection products are applied at flowering (to prevent flower rot, bunch trash colonization and latent infection); at pre-bunch closure (to prevent production of conidia on bunch trash); at veraison (to prevent infection by conidia or mycelium when berries begin to be susceptible); and at berry ripening (to prevent infection when berries are highly susceptible). Because a complete protection schedule based on interventions at all four growth stages is often unjustified [21,22], information and tools are needed to support decision-making by growers on when BBR control is likely to be effective. Together, agronomic features (including the susceptibility of the variety, trellis system, vineyard vigor, or the presence of wounds or cracks on the berry skin) represent a first risk factor to be considered [23,24,25]. Weather conditions represent a second risk factor; in the early season, infection severity increases with hours of wetness at temperatures near 20 °C [26,27,28,29]. From veraison to ripening, the risk of infection is highest at temperatures between 15 and 25 °C and also increases with hours of wetness or high relative humidity [19,24,27,29,30]. A mechanistic model was recently developed by González-Domínguez et al. [14] and was found to correctly predict disease risk based on weather conditions [31].

A third risk factor to be considered is the current level of *B. cinerea* establishment in the vineyard. Detection of *B. cinerea* in plant tissues is traditionally based on the direct plating of the tissues on agar media [32,33,34] or on microscopic examination of such tissues [7,9,12,16,35,36]. Latent infection in immature berries can be assessed by inducing tissue senescence by treating berries with paraquat or freezing [8,37]; latent infection in ripening berries can be assessed by incubating the berries under moist conditions [31]. All of these methods are time-consuming, lack sensitivity, often provide inconsistent results among assays and operators [32], and may be biased by the concomitant presence of other fungal species.

Continuous advances in DNA-based detection methods have provided fast, sensitive and reliable detection and quantification of fungal pathogens [38]. The newer DNA-based methods are generally superior to immunoassay methods such as the plate-trapped antigen enzyme linked immunosorbent assay (PTA-ELISA) [39] and to DNA hybridization methods such as the microfluidic chip assay (MCA) [40]. The PTA-ELISA and MCA assays are useful for studying latent infections of *B. cinerea* and early stages of the disease but are expensive and require complex operation and long processing times [39,40]. Molecular markers for specific polymerase chain reaction (PCR)-detection of *B. cinerea* were characterized by Rigotti et al. [41], which was followed by the development of a direct PCR assay for the detection of *B. cinerea* on pea-sized berries and receptacles [42]. Real-time quantitative PCR (qPCR) assays have also been developed for the detection and quantification of *B. cinerea* in developing grape berries [43], grape stamens and ripe berries [37,44,45,46], pea-sized berries and receptacles [47], and bunch trash [48]. Despite some successful applications, the above-mentioned PCR-based systems are not suitable for testing plant material at the field site (near the sampling location) [49,50,51]; therefore, they cannot be applied for routine, in-vineyard assessment of *B. cinerea*.

Loop-mediated isothermal amplification (LAMP) method [52], in contrast, enables the in-field detection of plant pathogens with portable devices [53,54,55,56]. Compared to PCR-based methods, LAMP amplification relies on a strand-displacing polymerase to amplify DNA under isothermal conditions [57]; LAMP-based methods, therefore, do not require thermal cycling. Moreover, LAMP enzymes are tolerant to substances that inhibit PCR reactions; hence, simple and rapid sample preparation methods, without DNA purification steps, are enough for LAMP assays [58,59]. LAMP assays have been used to detect *B. cinerea* strains that are resistant to benzimidazole fungicides and quinone outside inhibitors [60,61] and to detect *B. cinerea* in rose petals and pelargonium leaves [62], and in tomato and strawberry petals [63].

The aim of the present study was to evaluate the performance of a real-time LAMP-based method for the detection of *B. cinerea* in bunch trash and in immature and ripening berries. A simple method for the preparation of crude extracts was also developed and tested for the LAMP assay; if effective, this preparation method could be used as a part of a rapid in-field LAMP assay to evaluate the current establishment of *B. cinerea* in vineyards at critical stages for BBR management.

## 2. Results

### 2.1. Specificity and Analytical Sensitivity

**Figure 1:**
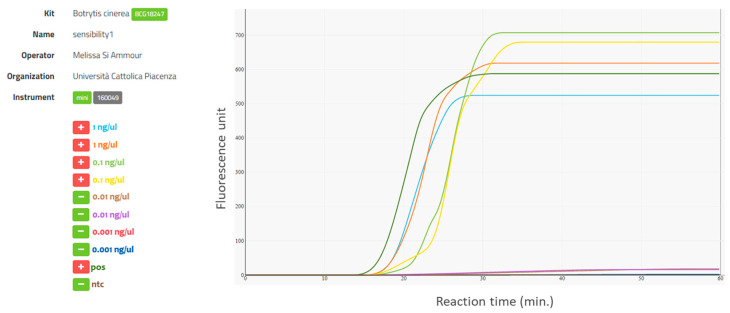
The real-time LAMP assay for *Botrytis cinerea* using 10-fold serially diluted DNA of *B. cinerea* (1.0 to 0.001 ng/µL per sample). LAMP amplification curves generated in an ICGENE mini (Cat.No.EBT 801) portable instrument. Amplification curves and sample labels are colored correspondingly. + indicates amplification, and - indicates no amplification; pos is the positive control consisting of *B. cinerea* DNA as template, and ntc is the control consisting of water as template.

When tested for its specificity to *B. cinerea*, the LAMP assay consistently amplified the DNA of *B. cinerea* but never amplified the DNA of non-target organisms (Table 1). Quantities of *B. cinerea* DNA at 1.0 ng/µL and 0.1 ng/µL were consistently amplified in all 8 replicate trials, while 0.01 ng/µL was amplified in only half of them, and 0.001 ng/µL was never amplified (Figure 1). Therefore, the analytical sensitivity of the LAMP assay for *B. cinerea* DNA was 0.1 ng/µL. Positive and negative controls always resulted in amplification and non-amplification of the *B. cinerea* DNA, as expected.

### 2.2. Evaluation of LAMP with Bunch Trash

The LAMP amplified the *B. cinerea* DNA in all bunch trash samples with colonization levels between 6 and 100%. The assay, however, amplified one-third of the samples that had not been artificially inoculated with *B. cinerea*.

### 2.3. Evaluation of LAMP with Berries

**Figure 2:**
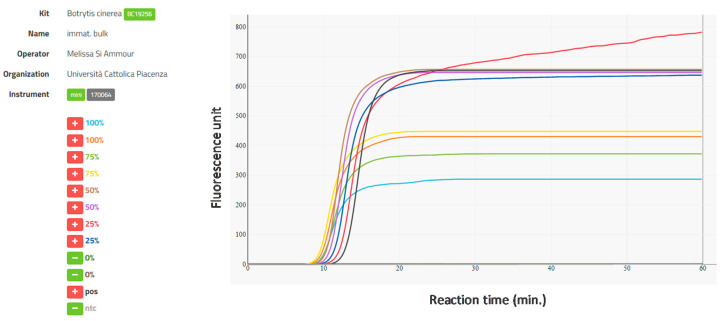
Amplification curves obtained with the real-time LAMP assay for *Botrytis cinerea* using immature berries in bulk samples with different levels of *B. cinerea* infection (0, 25, 50, 75, and 100%) and an ICGENE mini (Cat.No.EBT 801) portable instrument. Bulk-berry samples contained 4 berries per sample; for example, the 75% infection level contained 3 berries from bunches that had been inoculated with *B. cinerea* at flowering and 1 berry from bunches that had not been inoculated with *B. cinerea* at flowering. Amplification curves and sample labels are colored correspondingly. + indicates amplification, and - no amplification; pos is the positive control consisting of *B. cinerea* DNA as template, and ntc is the negative control consisting of water as template.

The LAMP amplified *B. cinerea* DNA from all individual immature and mature berries excised from clusters that had been artificially inoculated during flowering but did not amplify *B. cinerea* DNA from any berries from clusters that had not been inoculated with the fungus. Amplification also occurred in all of the bulk-berry samples containing 25, 50, 75, or 100% of inoculated berries, i.e., the LAMP assay was able to detect *B. cinerea* DNA in 1 infected immature and mature berry in a bulk sample of 4 immature berries in less than 20 min reaction time (Figure 2).

## 3. Discussion

In the present study, a real-time LAMP assay was evaluated in terms of specificity and sensitivity for the detection of *B. cinerea* in different grapevine tissues: bunch trash, immature berries, and ripening berries. A simple method of crude extract preparation, which can be conducted on-site with minimal laboratory equipment, was also developed for BBR diagnosis and early detection.

When *B. cinerea* plus 14 other fungi commonly present on grape vine bunches were tested, the LAMP assay was found to be specific for *B. cinerea* and was also found to be sensitive to a *B. cinerea* DNA quantity of 0.1 ng/µL. These results are consistent with previous studies in which LAMP assays specific for *B. cinerea* were developed and tested [62,63]. However, the analytical sensitivity determined in our assays was lower than the detection limit of 10^−3^ ng/µL reported by Duan et al. [63] and 6.5 pg reported by Tomlinson et al. [62]. The latter authors [62] estimated that the developed LAMP assay could detect the equivalent of 20 pathogenic cells. In addition, Mehli et al. [64] developed a TaqMan real-time PCR assay for *B. cinerea* with a detection limit of 1 pg, which corresponds to approximately three fungal cells. We therefore infer that our LAMP assay could detect the equivalent of approximately 300 pathogen cells. This analytical sensitivity level was sufficient to detect the lowest *B. cinerea* colonization levels tested in crude extracts of bunch trash (i.e., 6% of bunch trash colonized) and in immature and mature berries (25%, i.e., 1 of 4 berries infected in a bulk sample), with no amplification in *B. cinerea*-free material. Actually, the LAMP amplified some field-collected bunch trash samples that have not been artificially inoculated with *B. cinerea*; however, based on the overall results of this work, detection of the fungus in these samples may indicate that the bunch trash had been naturally colonized by *B. cinerea* in the vineyard before sample collection at full flowering rather than the assay having provided false positive detection. Therefore, the LAMP assay described here can be considered a sensitive tool for the on-site detection of *B. cinerea* in grape tissues.

LAMP methods have been developed to enable early on-site detection of plant pathogens from crude extract samples, including the detection of latent infections of *Plasmopara viticola* in grape leaves [65], grapevine phytoplasmas in crude leaf-vein homogenate [66], *Peronospora effusa* in symptomless spinach leaves [67], *Spiroplasma citri* in citrus leaves [68], and *Phytophthora infestans* in potato leaves [54], as well as the detection of plant viruses and viroids [69]. When coupled with spore trap systems, LAMP assays have been used for the detection and quantification of airborne pathogen inoculum, including the conidia of *Alternaria solani* and sporangia of *P. infestans* [70], and the conidia of *Magnaporthe oryzae* [56]. LAMP assays have been also developed for the quantification of airborne *Erysiphe necator* inoculum and have been evaluated for potential implementation by vine growers for the initiation of fungicide programmes [55,71]. LAMP has recently been applied for the detection of *B. cinerea* resistance to benzimidazole fungicides [60] and for the on-site detection of *B. cinerea* resistance to quinone outside inhibitors [61].

The LAMP assay tested in the current study has the potential to be used for improving the control of BBR in vineyards by enabling vineyard managers to assess the incidence of *B. cinerea* in some plant parts at critical growth stages. For instance, the decision to apply plant protection products at pre-bunch closure in order to reduce the production of conidia on bunch trash colonized by *B. cinerea* may depend on the colonization level; plant protection products could be omitted when the colonization is low [16,31,48]. The decision to apply products during berry ripening may depend on the incidence of latent infections that established in early growth stages; the first BBR foci in clusters are often caused by latent infections that reactivate after veraison and result in rotted berries, whose mycelium cause berry-to-berry infection and produce conidia for further infections [31]. Similarly, BBR may develop in grape bunches during transport or storage when apparently healthy grape berries with latent infections are harvested [31,37,72]. The assessment of *B. cinerea* colonization of bunch trash and of *B. cinerea* latent infections in young, ripening, and ripe berries may therefore improve decision making concerning BBR control. This assessment, however, is not part of the current practice, because the current laboratory methods for the detection of *B. cinerea* are complex, time consuming, and expensive.

The advantages of LAMP are numerous. LAMP is highly selective and sensitive, inexpensive, and rapid; in the current research, the average time required for processing a sample was <1 h, including the crude extract preparation and the LAMP reaction time. In addition, the LAMP assay is easy to perform.

It requires only (i) a set of four to six primers, with the addition of two Loop primers, that generate billions of DNA copies within 40 to 60 min and that increase the specificity, the sensitivity, and the speed of the reaction [56,73,74]; (ii) a strand-displacing DNA polymerase to amplify DNA; (iii) an instrument that maintains a constant temperature (LAMP is an isothermal process and does not require the expensive thermocyclers used for PCR), e.g., a portable battery-powered isothermal instrument such as that used in our experiments; and (iv) a portable battery-powered combined centrifuge/vortexer (combi-spin) for sample preparation, when necessary.

These features make LAMP particularly suitable for an in-field molecular testing system. Because LAMP relies on a strand-displacing polymerase to efficiently amplify DNA, or RNA sequences through combination with reverse transcription, it is less sensitive than PCR to potentially inhibitory substances (e.g., polysaccharides, polyphenols, phytoalexins, and lignin) present in complex samples such as plant tissues [58,66,75].

Practical use of LAMP for assessing the incidence of bunch trash colonized by *B. cinerea* and of berries with latent infections of *B. cinerea* should be based on detailed sampling and assessment via on-site LAMP assay. The current research has provided an introduction to such sampling and assessment, but further work is needed for the development and optimization of sampling and assessment protocols of LAMP in vineyard management.

In conclusion, the real-time LAMP assay has the potential to be used as a diagnostic tool for assessing *B. cinerea* colonization in bunch trash and latent infections of berries. The information provided would be highly valuable for decision-making regarding the control of BBR in vineyards. Early diagnosis and disease monitoring are key components of IPM because they support decision making and help avoid unjustified fungicide treatments and their negative effects [76,77].

## 4. Materials and Methods

### 4.1. Real-Time LAMP

#### 4.1.1. LAMP Kit

Real-time LAMP assays were performed using a commercial LAMP kit (*Botrytis cinerea* EBT-547, Enbiotech, Palermo, Italy). A reaction mixture (25 µL) was prepared by distributing 22 µL of LAMP mix, containing a strand-displacing polymerase, into 200 µL reaction tubes containing dried LAMP primers to which a template DNA was added (3 µL); the LAMP mix and primers were provided with the kit. Finally, mineral oil (30 µL) was added to the reaction tubes to prevent evaporation of the reagents. The reaction tubes were briefly centrifuged in a mini centrifuge, and immediately placed into the ICGENE mini (Cat.No.EBT 801) portable instrument (Enbiotech, Palermo, Italy), which measures fluorescence for real-time detection of the isothermal amplification of 12 samples simultaneously. LAMP amplifications were conducted at a constant temperature of 65 °C for 60 min. LAMP assays included a positive control and one negative control (no-template control) consisting of sterile-distilled water (provided with the kit). Positive and negative controls always resulted in amplification and non-amplification of the *B. cinerea* DNA, as expected.

#### 4.1.2. DNA Extraction

Purified genomic DNA was obtained from fresh mycelium of 15 fungal species (Table 1), which were used for the specificity and sensitivity assays. Fresh mycelium of each species was obtained by scrapping the surface of colonies grown on potato dextrose agar (PDA) for 10 days in an incubator at 20 °C and with a 12 h photoperiod. For *Plasmopara viticola* and *Erysiphe necator*, DNA was extracted from leaf pieces with sporulating lesions. Genomic DNA of the fungal isolates was obtained from about 100 mg of fresh mycelium from PDA or leaf discs. Each sample (100 mg) was placed in a 2 mL microcentrifuge tube, to which about 100 mg of glass sand (425–600 µm) and two glass beads (5 mm) were added. The samples were mixed with 500 µL of cetyl trimethylammonium bromide (CTAB) extraction buffer (2% CTAB, 100 mM Tris-HCl pH 8.0, 20 mM ethylenediaminetetraacetic acid [EDTA], 1.4 M NaCl, and 1% polyvinylpyrrolidone) and placed in a Mixer Mill MM200 (Retsch GmbH, Haan, Germany) for 1 min at 30 cycles/s. Subsequently, a CTAB DNA extraction procedure was followed as described by Si Ammour et al. [48]. A NanoDrop™ 2000 spectrophotometer (Thermo Fisher Scientific Inc., Waltham, MA, USA) was used to determine the yield and purity of the extracted DNA were determined, and the DNA concentration of each sample was adjusted to contain 10 ng of fungal DNA in a new microcentrifuge tube.

#### 4.1.3. Specificity

The most common grape pathogens and other fungal species frequently found in grapevines and in air samples from vineyards (Table 1) were used to test the specificity of the LAMP assay for the detection of *B. cinerea* in grape tissue. The *B. cinerea* strains, which belonged to the transposon genotypes *transposa* (T) or *vacuma* (V) [28], and the other fungi tested belong to the culture collection of the Università Cattolica del Sacro Cuore (UCSC), Piacenza (Italy), Department of Sustainable Crop Production of. *Plasmopara viticola* and *Erysiphe necator* isolates were sampled in 2017 and 2018 from USCC vineyard and were maintained by inoculation on grape plants (cv. Merlot) in a greenhouse. All specificity tests were conducted twice.

#### 4.1.4. Analytical Sensitivity

*Botrytis cinerea* DNA, obtained from PDA cultures as described earlier, was used to evaluate the sensitivity of the LAMP assay. The DNA was 10-fold serially diluted (from 1 to 0.001 ng/µL) in sterile-distilled water. The limit of detection (LOD) of the LAMP assay was defined as the minimum quantity of *B. cinerea* DNA from which consistent LAMP amplifications were obtained. The sensitivity tests were conducted four times, with two replicates each time. LAMP assays included a standard positive control consisting of *B. cinerea* DNA fragments (provided with the kit) and one no-template control (water control) consisting of sterile-distilled water (provided with the kit). Positive and negative controls always resulted in amplification and non-amplification of the *B. cinerea* DNA, as expected.

### 4.2. Evaluation of LAMP with Bunch Trash

#### 4.2.1. Plant Material and *B. cinerea* Inoculation

In 2017, bunch trash samples were collected in a vineyard in Castell’Arquato, Northern Italy (44°51′26.1′′ N 9°51′20.7′′ E, 400 m asl). The vines (*V. vinifera)* cv. Merlot were planted in 2007, known as a highly sensitive variety to *B. cinerea* [78,79]; the vines were trained using the Guyot system with 10-12 buds per cane, one cane per plant; 1.0 m within and 2.3 m between-row spacings. An integrated pest management (IPM) program [76] was followed to manage the vineyard, with no fungicides for the control of *B. cinerea*, with between-row grass, and with no irrigation. At full flowering (stage 65 [5]), bunch trash was sampled from 50 random clusters by shaking them carefully inside paper bags; bunch trash consisted of calyptras, stamens, unset flowers, and aborted fruitlets. Bunch trash was carried to the laboratory in a cool bag at 5 °C, and instantly desiccated at 35–40 °C for 72 h, weighed, and divided into 1 g (dry weight) samples, which were then kept at room temperature.

For inoculum preparation, 10-day-old cultures grown on PDA in Petri dishes of *B. cinerea* (isolate 213T) were used to obtain a conidial suspension as described earlier. The dishes were flood with sterile-distilled water and gently rubbed on the surface with a sterile rod and the obtained conidial suspension was passed through two layers of autoclaved gauze. The number of conidia was determined using a hemocytometer and the concentration was adjusted to 10^5^ conidia/mL.

For inoculation, each 1 g bunch trash sample was spread on autoclaved filter paper on the bottom of a Petri dish (60 mm in diameter); a micropipette was then used to uniformly inoculate each sample with 1 mL of the *B. cinerea* conidial suspension. One ml of sterile-distilled water was added to other bunch trash samples as a negative (non-inoculated) control. Petri dishes with either inoculated or non-inoculated bunch trash samples were placed in a growth chamber at 20 °C, in the dark to favor conidial germination, mycelial growth, and bunch trash colonization. After 18 h in the growth chamber, the bunch trash samples were placed in a laminar flow hood at room temperature and left to dry for 2 h.

#### 4.2.2. Preparation of Crude Extracts from Bunch Trash

Bulk samples of bunch trash were prepared with different levels of *B. cinerea* colonization (0, 6, 12, 25, 50, 75, and 100%), which were obtained by blending inoculated and non-inoculated bunch trash samples. Crude extracts were obtained from each of the seven bunch trash colonization levels in three replicate samples (0.1 g each). Samples were placed in a 2 mL microcentrifuge tube with 1 mL of extraction buffer (provided with the LAMP kit); the samples were shaken in a vortex apparatus for 10 s and then kept for 10 min at room temperature. The tubes were centrifuged for 10 s in a minicentrifuge, and the crude extract was diluted 1/10 in sterile-distilled water. The diluted crude extract was then used as a template in the LAMP assays. The experiment was conducted three times. LAMP assays included a standard positive control consisting of *B. cinerea* DNA fragments (provided with the kit) and one no-template control (water control) consisting of sterile-distilled water (provided with the kit). Positive and negative controls always resulted in amplification and non-amplification of the *B. cinerea* DNA, as expected.

### 4.3. Evaluation of LAMP with Berries

#### 4.3.1. Plant Material and *B. cinerea* Inoculation

Grape plants in pots (20 plants, cv. Merlot) were grown in the experimental vineyard of the UCSC campus in 2018 and 2019. At growth stages 62 and 65 (beginning and full flowering, respectively), 30 inflorescences (still attached to the plants) were uniformly sprayed with a conidial suspension of *B. cinerea* (10^5^ conidia/mL) prepared as previously described. The inoculated inflorescences were sealed in polyethylene bags immediately after inoculation (to maintain moisture and favor the infection by *B. cinerea*) for 24 h. At the same time, 30 inflorescences at growth stages 62 and 65 (still attached to the plants) were not inoculated but were sprayed with a commercial fungicide containing fludioxonil (25%) and cyprodinil (37.5%) (Switch; Syngenta Crop Protection), at 0.8 g/l, to prevent natural infection. Both inoculated and non-inoculated inflorescences were kept in paper bags during fruit development to prevent natural infections by *B. cinerea*.

At the growth stages 79 (end of fruit development) and 89 (berries ripe for harvest), both inoculated and non-inoculated bunches were collected, washed using tap water, and surface sterilized, by immersion in a sodium hypochlorite solution (30%) for 1 min, and rinsed three times in sterile-distilled water, to remove epiphytic microflora including *B. cinerea* which may be present on the berry surface. Bunches collected at growth stages 79 and 89 were used to evaluate the LAMP detection of latent infections of *B. cinerea* in immature and mature berries, respectively, in both single-berry and bulk-berry assays (described in the next section).

For the assay with immature berries, berries were excised with the pedicel from the clusters that had been or had not been inoculated during flowering; the former but not the latter were expected to have latent infections. To confirm the presence of latent infections, a sub-sample of 20 freshly collected berries were cut in half using a sterile scalpel under a laminar flow hood, and the halves were placed on PDA in Petri dishes with the cut surface down. The dishes were sealed and incubated at 20 °C with a 12 h photoperiod to favor fungal growth on the medium. Dishes were inspected daily to assess the incidence of berries showing the development of *B. cinerea* colonies. No fungal growth was observed from berries excised from non-inoculated bunches, while typical *B. cinerea* colonies grew from 65% of the berries from inoculated bunches.

For the assay with mature berries, berries were excised with the pedicel from the clusters that had not been inoculated during flowering. These berries, which were expected to be free of infection, were rinsed three times with sterile-distilled water, surface-sterilized, to remove epiphytic microflora including *B. cinerea* which may be present on the berry surface, by immersion in a sodium hypochlorite solution (30%) for 1 min, rinsed three times in sterile-distilled water, and placed separately on a layer of filter paper on the bottom of Petri dishes (90 mm in diameter; four berries per dish). The pedicel was removed using a sterile scalpel, and each berry was inoculated by depositing a 10 µL drop of *B. cinerea* inoculum (10^5^ conidia/mL; prepared as described earlier) on the pedicel wound. Other berries were inoculated with a drop of sterile water and represented the non-inoculated control. The Petri dishes were sealed with Parafilm^®^ M (Merck Life Science S.r.l., Milano, Italy) and were placed in an incubator at 20 °C for 24 h to favor conidial germination and berry infection. One day after inoculation with either *B. cinerea* or sterile water, all berries were symptomless and were used for the preparation of crude extract (described in the next section).

#### 4.3.2. Preparation of Crude Extracts from Berries

Individual berries (either immature or mature) were cut in half using a sterile scalpel. For single-berry assays, berries were processed individually. For bulk-berry assays, berries were grouped (4 berries per group) to obtain the following expected levels of *B. cinerea* infection: 0, 25, 50, 75, and 100%. For the bulk-berry assays with immature berries, for example, the 75% infection level contained 3 berries from inoculated bunches and 1 berry from non-inoculated bunches. For the bulk-berry assay with mature berries, for example, the 75% infection level contained or 3 wounded berries that had been inoculated with the fungus and 1 wounded berry that had been inoculated with sterile water. Individual berries were placed in a 2 mL microcentrifuge tube with 1 mL of extraction buffer (provided with the LAMP kit), and bulk-berry samples were placed in a 50 mL centrifuge tube with 3 mL of extraction buffer. Both single-berry and bulk-berry samples were shaken using a vortex apparatus for 10 s and kept at room temperature for 10 min. The tubes were then centrifuged for 10 s in a microcentrifuge, and the crude extract was diluted 1/10 in sterile-distilled water. The diluted lysate was used promptly as a template in the LAMP assays. The experiment was conducted three times, with two replicate samples. LAMP assays included a standard positive control consisting of *B. cinerea* DNA fragments (provided with the kit) and one no-template control (water control) (provided with the kit) consisting of sterile-distilled water, which resulted in amplification and non-amplification of the *B. cinerea* DNA, respectively.

## 5. Patents

Patent licenses WO 00/28082, WO 01/34790, WO 01/77317, WO 02/24902, and WO 01/34838, owned by Eiken Chemical Co., Ltd., Tokyo, Japan.

Patent for industrial invention n. 0001425753 dated 09.11.2016, extended with priority in Europe with question no. EP15179273.6 by Bionat Italia S.r.l., used under license by Enbiotech S.r.l.

## Figures and Tables

**Table 1:** List of isolates used for specificity tests of the real-time loop-mediated isothermal amplification (LAMP) assay.

Genus and Species	Isolate Code	LAMP Result ^a^
*Alternaria alternata*	5	-
*Alternaria* sp.	23	-
*Aspergillus flavus*	4	-
*Aspergillus niger*	A1	-
*Botrytis cinerea*	213T and 351V	+
*Erysiphe necator*	FP ^b^ 2017	-
*Guignardia bidwellii*	Q15	-
*Monilia laxa*	11	-
*Penicillium* sp.	2	-
*Phomopsis viticola*	Pho-6	-
*Plasmopara viticola*	FP 2018	-
*Rhizopus* sp.	26	-
*Rhizopus stolonifer*	MUCL38013	-
*Sclerotinia sclerotiorum*	22	-
*Stemphylium* sp.	14	-
*Vitis vinifera*		-

^a^ + indicates amplified, and - indicates not amplified. ^b^ FP: field population and year of collection.
